# Electro-optically modulated lossy-mode resonance

**DOI:** 10.1515/nanoph-2021-0687

**Published:** 2021-12-15

**Authors:** Mateusz Śmietana, Bartosz Janaszek, Katarzyna Lechowicz, Petr Sezemsky, Marcin Koba, Dariusz Burnat, Marcin Kieliszczyk, Vitezslav Stranak, Paweł Szczepański

**Affiliations:** Warsaw University of Technology, Institute of Microelectronics and Optoelectronics, Koszykowa 75, 00-662 Warsaw, Poland; University of South Bohemia, Branisovska 31, 37005 Ceske Budejovice, Czech Republic; National Institute of Telecommunications, Szachowa 1, 04-894 Warsaw, Poland

**Keywords:** electro-optical modulation, label-free sensing, lossy-mode resonance, magnetron sputtering, optical fiber sensor, transparent conductive oxides (TCOs)

## Abstract

Sensitivity, selectivity, reliability, and measurement range of a sensor are vital parameters for its wide applications. Fast growing number of various detection systems seems to justify worldwide efforts to enhance one or some of the parameters. Therefore, as one of the possible solutions, multi-domain sensing schemes have been proposed. This means that the sensor is interrogated simultaneously in, e.g., optical and electrochemical domains. An opportunity to combine the domains within a single sensor is given by optically transparent and electrochemically active transparent conductive oxides (TCOs), such as indium tin oxide (ITO). This work aims to bring understanding of electro-optically modulated lossy-mode resonance (LMR) effect observed for ITO-coated optical fiber sensors. Experimental research supported by numerical modeling allowed for identification of the film properties responsible for performance in both domains, as well as interactions between them. It has been found that charge carrier density in the semiconducting ITO determines the efficiency of the electrochemical processes and the LMR properties. The carrier density boosts electrochemical activity but reduces capability of electro-optical modulation of the LMR. It has also been shown that the carrier density can be tuned by pressure during magnetron sputtering of ITO target. Thus, the pressure can be chosen as a parameter for optimization of electro-optical modulation of the LMR, as well as optical and electrochemical responses of the device, especially when it comes to label-free sensing and biosensing.

## 1 Introduction

Rapid and reliable biosensing solutions are highly desired nowadays [1]. Such devices, especially when they make point-of-care testing possible, are mainly expected to identify pathogen outbreaks [2]. High selectivity, wide range, low detection limit, short response time, small size and low fabrication costs are among highly anticipated parameters of the biosensor [3]. To satisfy all these requirements and additionally limit false positive results, a set of sensors together with advanced data processing is typically applied [4]. That is why, sensors offering multiple sources of information, at best received from the same spot and at the same time, but in different domains, such as optical, electrical, mechanical, etc., are of high interest. A couple of dual-domain sensors, i.e., combing electrochemistry (EC) and optical spectroscopy, has already been reported [5]. To receive simultaneously electrical and optical readouts surface of the sensor must be both electrically conductive and susceptible to optical effects that are influenced by a measurand. For these purposes the most often explored phenomenon, among many others, is the surface plasmon resonance (SPR), where excitation and propagation of plasmon at a surface of thin gold film is influenced by optical properties of an analyte covering the surface [6]. In the dual-domain configuration the same gold film plays a role of an electrode in EC setup, where the charge transfer is influenced by electrical properties of the analyte at the surface. In certain conditions, e.g., when the sensor surface is functionalized for label-free sensing, only specific chemical or biological targets bind to the surface forming a film that disturbs both plasmons and charge transfer [7]. Besides gold, also a couple of other materials and physical effects allow for simultaneous optical and electrical/EC interrogations. Such planar sensing solutions as silicon ring resonators [8] and indium tin oxide (ITO) coated waveguides [9] can be pointed out as alternatives to gold-based SPR. Some other approaches based on optical fibers instead of planar structures were also reported, such as e.g., gold-coated tilted fiber brag gratings [10], ITO-coated long-period gratings [11], and ITO-coated fiber probes for spectroelectrochemical applications [12], [13], [14]. Optical-fiber-based sensing solutions additionally offer, e.g., possibility of remote and multiparameter sensing, resistance to electromagnetic interference, and compactness of the device.

By employing an additional domain, the sensing approaches are more likely to: operate at wider variety of distances from the sensor surface [15]; cross-verify the readouts making the sensor highly reliable [16, 17]; or enhance the sensor functionality [18, 19]. Interactions between the domains typically disturb cross-verification capability, but simultaneously may be employed reaching higher sensitivity and widen the range. The electro-optical interactions originate mainly from change in distribution of charge carriers in the sensor material, what in turn is followed by modulation of optical properties, i.e., real (*n*) and imaginary (*k*) part of refractive index (RI) of the material. Different effectiveness of the interactions has been reported for metallic film, e.g., gold [10, 20], and semiconductors, e.g., ITO and Si [21, 22]. Among semiconductors, ITO as one of transparent conductive oxides (TCOs) is of a particular interest due good EC performance.

In our previous works we have proposed a dual-domain (EC and optical) sensor based on the lossy-mode resonance (LMR) effect taking place on an optical fiber coated with ITO thin film [23]. The ITO surface may additionally undergo chemical and/or biological functionalization which makes such label-free sensing device highly selective. For specific LMR sensors configuration identification of various biomolecules [24] and chemical compound [25] even at femtomolar concentration is possible [26]. The LMR-based dual-domain label-free sensing of proteins [16, 19], and antibodies [17] has also been reported. To obtain LMR, the film must be thick enough to guide a mode and *n* of the film must be positive and higher in magnitude than both its *k* and *n* of the surrounding medium [27]. It means that the ITO film has to be optimized to display LMR in a spectral response. This is especially valid for structures offering high sensitivity to a selected measurand. In our works ITO films were fabricated using low-temperature plasma assisted deposition employing magnetron sputtering (MS) driven either at radio frequency (RF) or with high-power impulse MS (HiPIMS) [28]. MS allows precise control over the deposition parameters, such as gas composition, power, heating, deposition pressure and other. Subsequently, these parameters determine film properties, such as RI, thickness and electrical resistivity [29]. Our former works clearly show that the tailoring of ITO films plays a key role it sensing properties of LMR-based devices, in both optical and EC domains [17]. Moreover, in contrast to e.g., Au-based EC-SPR, depending on ITO deposition conditions optical response of the sensor can be modulated with EC stimulus, what can be considered as a source of additional information about the interactions taking place at the sensor surface [16]. However, despite in-depth discussion on elementary EC processes taking place at the ITO-LMR surface [30], the phenomenon responsible for advanced LMR-based electro-optical detection has not been studied.

This work aims to unveil the elementary processes that are responsible for electro-optical modulation of the ITO-LMR sensors and to bring theoretical foundations for fabrication of these dual-domain sensors. For these reasons, technological experiments followed by optical and EC measurements were supported by numerical calculations. We show that a charge carrier density in ITO is mainly responsible for electro-optical modulation effectiveness and it needs to be tailored at the stage of ITO deposition to reach dual-domain interrogation capability.

## 2 Experimental details

### 2.1 Numerical analysis

**Figure 1:**
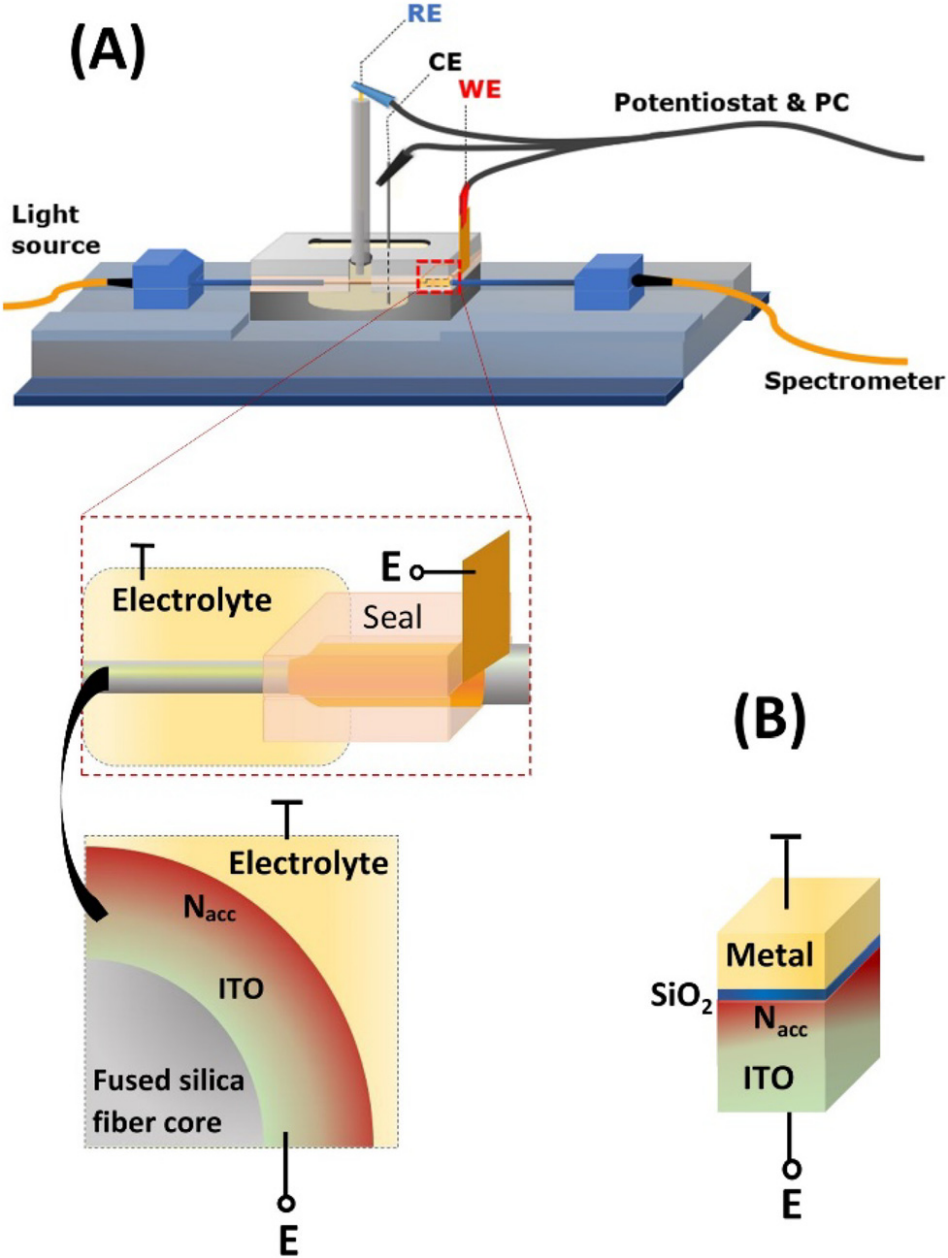
Schematic representation of (A) experimental setup for combined optical and EC analysis with the investigated ITO-coated optical fiber structure and part of its cross-section. In (B) electrically equivalent model of the optical structure is schematically shown, where a monolayer of SiO_2_ was introduced on ITO to represent oxidation of the film surface.

In this work, double-domain (optical and EC) approach that utilizes both, the optical and electrical advantages of ITO thin films is assumed. Figure 1 shows the schematic of an interface between the domains for optical fiber structure (Figure 1A) and its equivalent electrical model (Figure 1B). Considered simplified electrical model of the sensor corresponds to a metal–insulator–semiconductor (MIS) capacitor with a single Cu electrode and concentration of free charge carriers (electrons) of ITO (*N*) ranging from 3e19 cm^−3^ to 3e20 cm^−3^. This range is in agreement with experimental results presented elsewhere [31, 32]. The thickness of ITO film was set to 250 nm which corresponds to microscopic analysis of the ITO films on the surface of an optical fiber allowing for receiving LMR [33]. The ITO thickness was optimized using *in situ* spectrum monitoring approach during the deposition to attain 1st order LMR in the spectral range of operation. Moreover, a monolayer (0.2 nm) of SiO_2_ representing oxidized surface of the material, was introduced in the electrical model at the ITO-metal interface to make the MIS capacitor assumption valid. Presence of this layer in the model originates from the fact that ITO is often suboxidized and its surface becomes fully stoichiometric when exposed to standard environmental conditions [17]. In an experimental setup shown schematically in Figure 1A, the ITO-LMR sensor is immersed in an electrolyte (represented by metal in the electrical model), and the ITO film plays a role of a working electrode biased by the potential *E*. When *E* changes, as it happens in EC setups, the charge carrier density at the ITO surface follows. In this work the mean density of accumulated charge carriers *N*_acc_(*E*) has been calculated according to the procedure reported in [34]. Next, optical properties at the surface of ITO, namely *n*_acc_ and *k*_acc_ have been calculated using well-established Drude model of scattering of electrons by relatively immobile ions [35] according to Eq. (1), where *ω* is the field angular frequency, *ε*_∞_ = 3.9 is the high-frequency permittivity, *Γ* = 2.9e15 s^−1^ is the electron collision frequency [36], and *ω*_p_ represents the plasma oscillation resonance. The *ω*_p_ is determined by the ITO doping and defined by Eq. (2), where *N*_acc_ is the charge carrier density, *e* is the elementary charge, *ε*_0_ is the vacuum permittivity and *m*_eff_ = 0.35*m*_0_ [37] is the effective mass of electron.

(1) $${\left(n_{\text{acc}}+ik_{\text{acc}}\right)}^{2}=\epsilon_{\text{acc}}=\epsilon_{\infty }-\frac{\omega_{\text{p}}^{2}}{\omega^{2}+i\omega \Gamma }$$

(2) $$\omega_{\text{p}}=\sqrt{\frac{N_{\text{acc}}\cdot e^{2}}{\epsilon_{0}m_{\text{eff}}}}$$

Next, the calculated *n*_acc_ and *k*_acc_ were used to describe a layer with a thickness corresponding to accumulation depth (*d*_acc_). For calculation of the spectral response of the ITO-LMR structure, whole ITO layer, i.e., bulk and the accumulation layer, has been considered as a single effective layer of 250 nm and described by effective RI, i.e., *n*_eff_ and *k*_eff_. For this purpose, an optimization-based approach for extraction of optical constants has been formulated inspired by Male method [38]. The approach is based on a transfer matrix model suitable for description of electromagnetic response of plane–parallel media. Finally, spectral response of the ITO-LMR structure for different *E* was calculated using a numerical model described in [39], where silica glass and water were assumed as substrate and external medium, respectively.

### 2.2 ITO-coated optical fiber fabrication

Reactive magnetron sputtering of ITO was carried out in a high-vacuum chamber capable to attain the back-down pressure of 1e−6 Pa and assure the high purity of the deposition. The configuration of the deposition system is given in detail in [17]. A commercial planar 3-inch magnetron equipped with a compound ITO target (In_2_O_3_/SnO_2_ of composition 90/10 wt% and a purity of 99.99%) was installed in the stainless-steel vacuum chamber. Samples were placed 15 cm away from the sputtering target. For the fabrication of the samples approx. 15 cm long multimode polymer-clad silica fibers with 400/730 μm (core/cladding) diameter were used. For each fiber its central part (ca. 25 mm) of the cladding was removed and after ITO deposition it served as an active sensing area. The samples were rotated during the deposition to receive a uniform ITO coating around them. The sputtering was driven using RF COMET Cito 1310 supply with frequency 13.56 MHz and transmitted power of 150 W. Argon (purity of 99.999%) flow was kept constant at 100 sccm. Pressure (*p*) and deposition time (*t*) were tuned in range 0.1–1 Pa and 100 to 29 min, respectively, to tailor the highest optical sensitivity corresponding to the first attenuation band of LMR [27]. The range of *p* was found during initial experiments and recognized as appropriate for maintaining effective sputtering processes. It is worth noting that the deposition parameters were optimized to tailor ITO properties for dual-domain interrogation with respect to the system geometry and conditions of sputtered target. The *in situ* and online optical spectrum monitoring during the deposition was employed to prepare sensitivity-wise optimized structures, i.e., 1st order LMR was sought.

### 2.3 Measurements

Optical transmission of the ITO-coated fiber structure was interrogated in the range *λ* = 350–1050 nm using Ocean Optics HL-2000 white light source and Ocean Optics USB4000 spectrometer. Although the spectral response of the LMR structure is polarization dependent, the setup was kept simple, without any polarization managing devices. In general, the magnitude and location of the minimum in the spectral response of the sensor differs for each polarization. However, with the unpolarized light source and the multimode optical fiber the more pronounced resonance dip may be observed and traced, since contributions of the polarizations superimpose with one another [40]. Therefore, the setup was used as a simple, robust, and experimentally proved concept. First, the sensitivity of the samples to changes of external RI (*n*_ext_) was determined by immersion in water/glycerin solutions of different concentrations *n*_D_ = 1.3330–1.4200 RIU. Rudolph J57 automatic refractometer was used to verify *n*_ext_ of the solutions. Next, each sample was installed in the EC cell, as shown in Figure 1A, where it acted as a working electrode. A platinum wire and an Ag/AgCl 0.1 M KCl were used as a counter and a reference electrode, respectively. The experimental setup has been described in details in [33]. The EC performance of the films was tested in cyclic voltammetry (CV) configuration using a PalmSens Emstat3+ potentiostat/galvanostat controlled by a PSTrace 5.4 software. All the measurements were performed at room temperature in 0.1 M KCl (POCh, Poland) solution containing 1 mM 1,1′-ferrocenedimethanol (Acros Organics) as a redox system. All CVs were obtained at *E* reaching from −1 to 1 V versus Ag/AgCl electrode at a scan rate of 20 mV/s. Optical transmission (*T*) was traced in parallel to EC measurements. In-house developed software based on Matlab was used to synchronize optical and EC data.

## 3 Results and discussion

### 3.1 Influence of charge carrier density on the modulation of ITO’s optical properties

**Figure 2:**
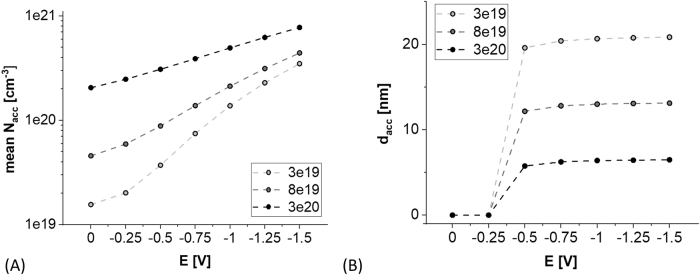
*E*-induced evolution of accumulation layer where (A) and (B) shows mean charge carrier density and thickness of the layer, respectively, for initial bulk carrier density *N* reaching 3e19, 8e19, and 3e20 cm^−3^.

ITO is an n-type semiconductor in which due to changes in composition and molecular structure the density of free charge carriers (*N*) can be tuned in a wide range, typically from 1e19 to 1e20 cm^−3^ [41]. Additionally, *N* can be locally modified by electric field *E* [42]. Negative and positive *E* induces, respectively, accumulation and depletion of majority carriers at the ITO surface. The more negative *E* becomes the higher the density of accumulated carriers *N*_acc_ and the deeper (higher *d*_acc_) into the surface the accumulation spreads. Figure 2A shows the dependence of mean *N*_acc_(*E*) for different *N*. It can be seen that ability to form the accumulation layer is determined by *N*, and for its lower values (order of 1e19 cm^−3^) the *d*_acc_ can reach tens of nanometers (Figure 2B) with an average increase of *N*_acc_ by over an order of magnitude (Figure 2A). For EC applications of ITO, *N* plays a crucial role. The Fermi level occurring in the forbidden energy gap of a semiconductor is influenced by the doping. Enhanced *N* brings the Fermi level up, what may encourage part of the electrons to contribute to the EC charge exchange between the electrolyte and the electrode.

**Figure 3: j_nanoph-2021-0687_fig_003:**
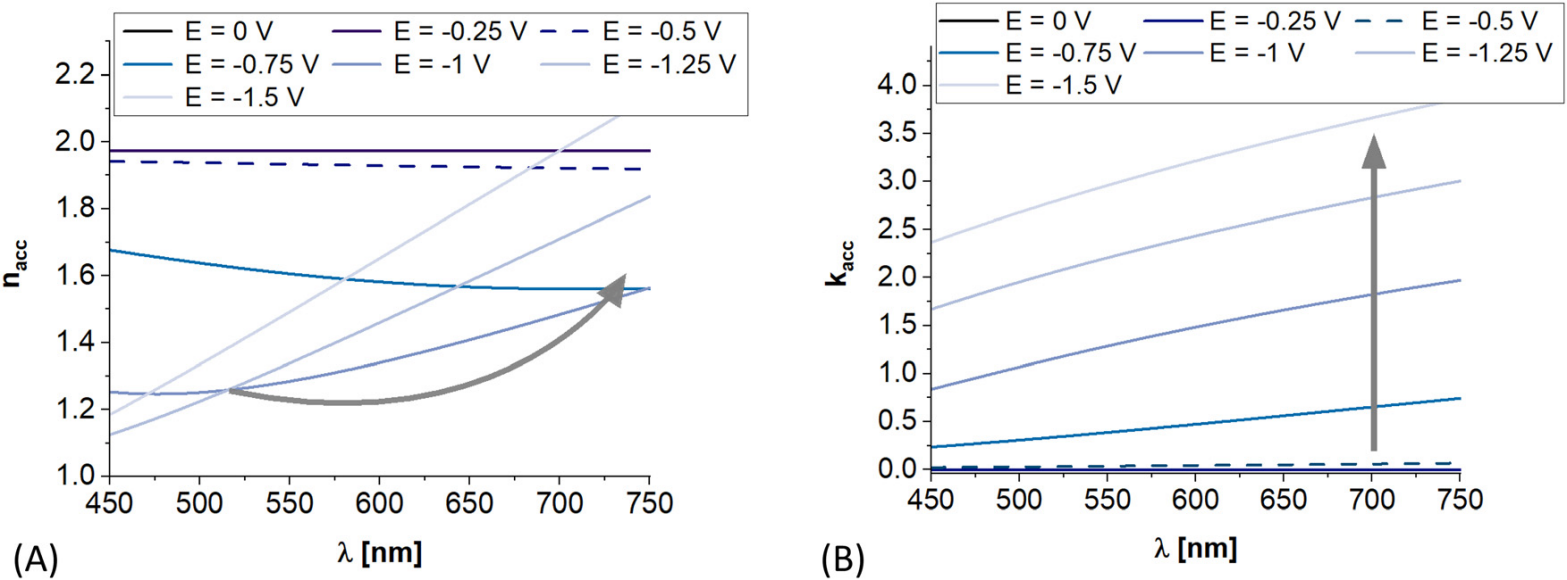
*E*-induced evolution of an averaged (A) refractive index and (B) extinction coefficient in the accumulation layer. The results are shown for *N* reaching of 3e19 cm^−3^ where accumulation layer of ∼20 nm appears for *E* < −0.5 V (see Figure 2).

Modulation of *N*_acc_ is followed not only by changes of electrical, but also optical properties of ITO [21]. Figure 3 shows analytical estimation of how significant the changes of RI of ITO in the accumulation layer can be. For *N* = 3e19 cm^−3^ the changes are effective when *E* < −0.5 V. In the analyzed case, in UV–Vis spectral range *n*_acc_ may decrease and *k*_acc_ may increase by over 0.5 RIU and 1 RIU, respectively, and reach over *d*_acc_ = 20 nm in depth of the material. Further decrease in *E* induces rise of *N*_acc_ and shift of the Fermi level toward metallic-like character of the ITO layer. This is in particular indicated by the turn and rise of *n*_acc_(*λ*) as *E* becomes more negative < −0.5 V (Figure 3A).

**Figure 4:**
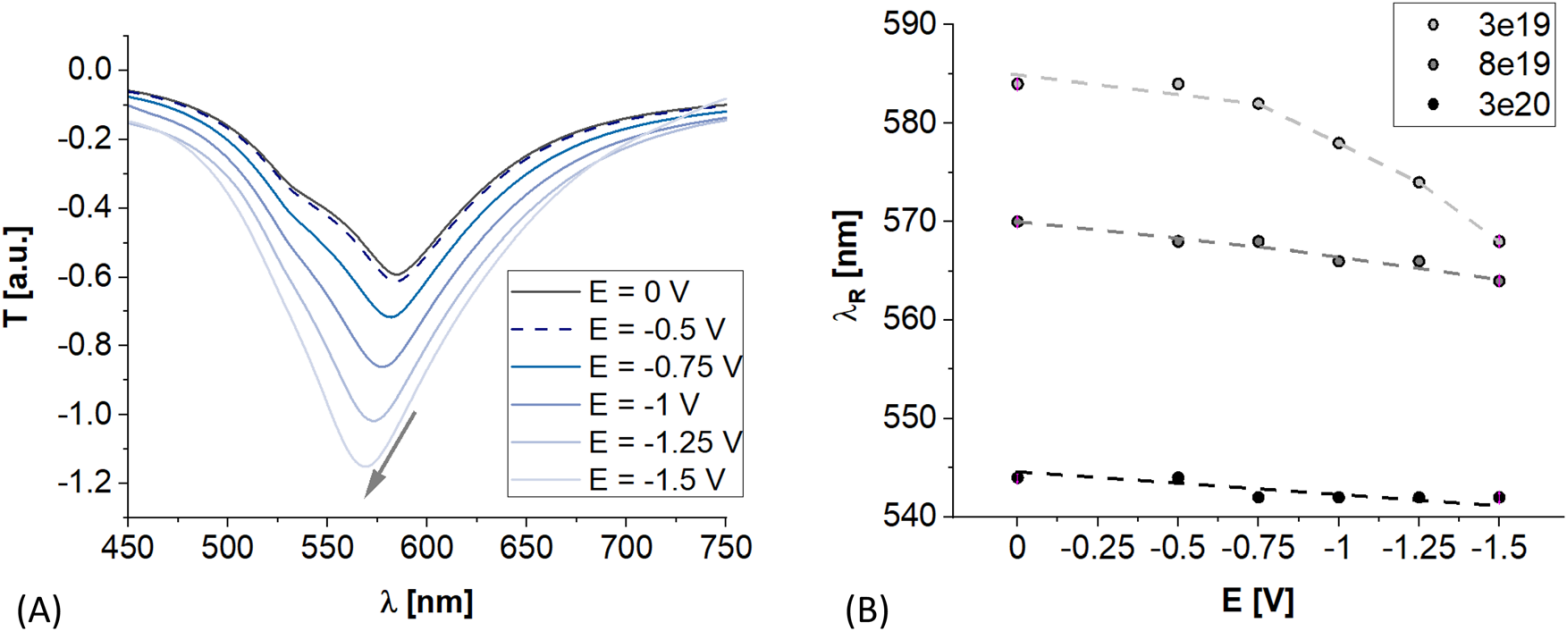
(A) *E*-induced evolution of ITO-LMR spectral response when charge carrier density reaches 3e19 cm^−3^ and (B) LMR wavelength shift with *E* when *N* ranges from 3e19 cm^−3^ to 3e20 cm^−3^.

Since *E* influences *n*_acc_ and *k*_acc_ (Figure 3), the ITO-LMR structure undergoes the *E*-induced changes which manifest themselves in LMR response (Figure 4). The LMR follows the decrease of *E*, i.e., the lower *E* is applied the deeper LMR is observed and corresponding wavelength *λ*_R_ experiences a blueshift. This is clearly visible in Figure 4A for lower *N*, i.e., *N* = 3e19 cm^−3^, when *E* drops below −0.5 V. The spectral changes with *E* are significantly less effective for higher *N* (Figure 4B). These results qualitatively corresponds well with experimental studies reported in [16], and indicate that for formerly shown experimental results initial *N* may be below 3e19 cm^−3^. Following values of *N* of ITO films indicated in [43], as low as 1e19 cm^−3^ can be achieved for this material. Moreover, a significant difference between numerical and experimental analysis must be noted, i.e., numerical data are received at stationary conditions, while EC measurements are performed at dynamically changing (scanning) *E*. The lower is the scan rate, the higher LMR spectrum modulation is recorded [30]. It must be also noted that the *λ*_R_ decreases with *N* (Figure 4B), what is important for discussions on optical properties of ITO included in Section 3.2.

The results of numerical analysis (Figures 2 [Fig j_nanoph-2021-0687_fig_003]–4) indicate capability of electrical modulation of ITO-LMR properties. It is shown that charge carrier density, the depth of accumulation zone, and the LMR spectral shift are highly influenced by initial bulk density of charge carriers (free electrons in the case of ITO). Higher accumulation of carriers corresponds to higher electrical conductivity. In contrast, significant *λ*_R_ shifts with *E* can be achieved when *N* is low. Thus, in the next section, capability for tuning ITO properties at its deposition state will be discussed in detail.

### 3.2 Tuning ITO properties at the stage of its deposition

It has already been shown that properties of ITO film deposited by means of magnetron sputtering depend on many process parameters that include deposition power, used electric field (DC, RF, impulse DC), process gas composition, base and working pressure, distance between the sample and the target, as well as substrate temperature and deposition time [17, 29]. Since ITO is widely applied, an impact of these parameters on optical and electrical properties is crucial for certain application and the films have been intensively studied during the last two decades. In our earlier work devoted to ITO-LMR sensors [33], capability to change optical properties of ITO and corresponding sensors response by deposition pressure (*p*) has been shown. The encouraging results of that work allow us to set the *p* to be the tuning parameters also for the study presented here.

**Figure 5:**
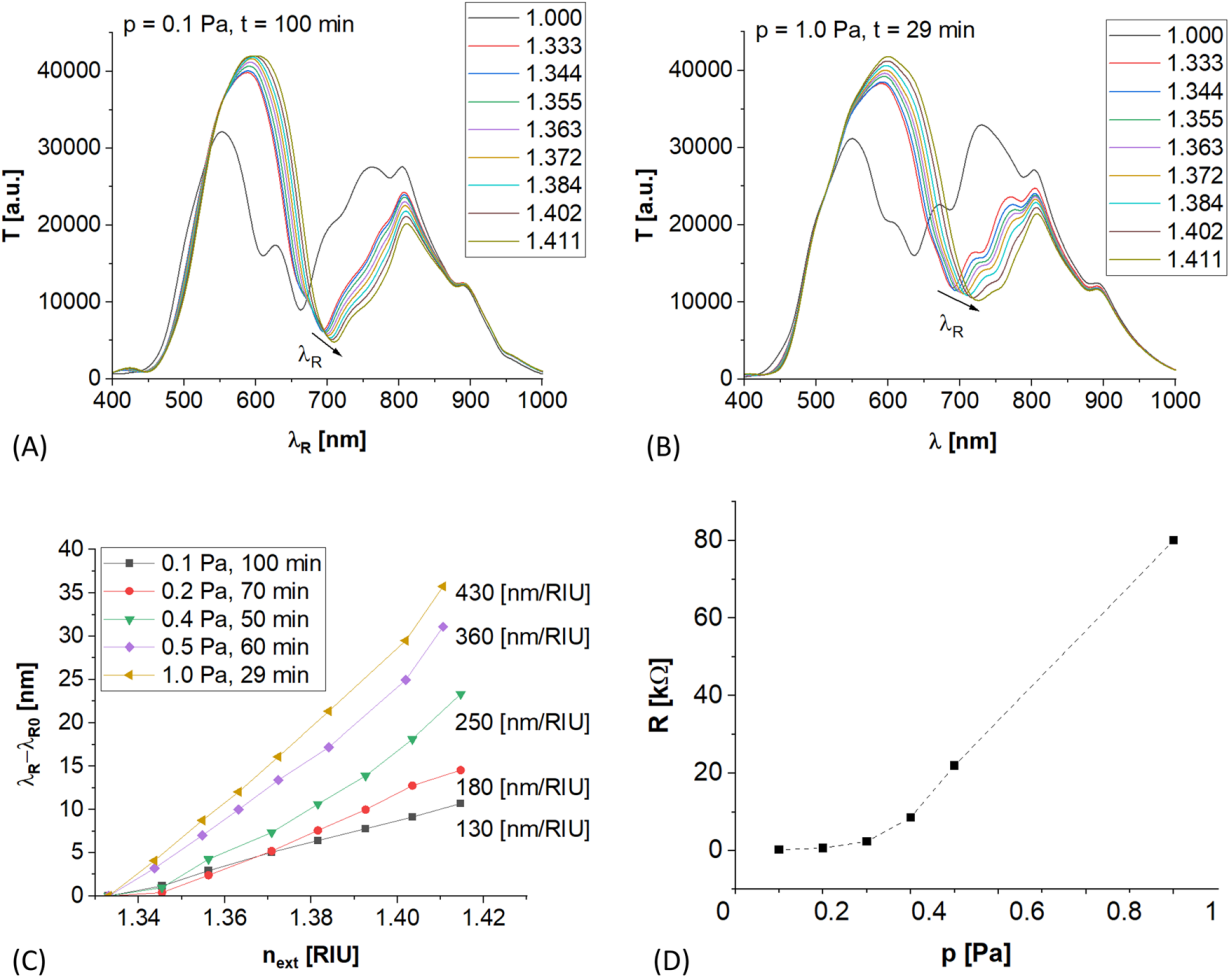
Evolution of ITO-LMR sample properties with *p*. Examples of similar LMR spectral patterns in response to *n*_ext_ varying from 1 up to 1.42 RIU received at different ITO deposition *p* and *t* are shown in (A) and (B). In (C) is shown relative shift of the *λ*_R_ with *n*_ext_ for selected sets of *p* and *t*. The *λ*_R0_ is the *λ*_R_ at *n*_ext_ = 1.3330 RIU for each set of *p* and *t*. In (D) is shown relation between resistance (*R*) of 25-mm-long active part of ITO-LMR structure and the *p*.

In Figure 5A and B spectral responses for ITO-LMR samples received at different set of *p* and *t* are shown. It has to be noted that the film thickness (*d*) is another variable parameter that was optimized by *t*, i.e., *t* was always adjusted to receive possibly similar spectral pattern (*λ*_R_ at ∼650 nm for *n*_ext_ = 1 RIU). The visible spectral range has been selected due to widely available instrumentation for interrogation in this range. In general, it is possible to receive similar spectral responses for different *p*, but *t* needs to be adjusted since the deposition rate (and subsequently *d*) follows the *p*. This effect is well-known and explained by a mean free path of sputtered atoms influenced by enhanced collisions in plasma at elevated *p* [44]. On top of the deposition rate, *p* has an impact on optical properties of the films, in particular its *n* and *k* [29, 33]. Investigations reveal that both the optical parameters are of crucial importance and strongly dependent on deposition process. In Figure 5C the spectral shifts, that corresponds to the RI sensitivity, are shown for different *p*. The *λ*_R_ shift follows the *p* – the RI sensitivity increases by over 150% when *p* increases from 0.1 to 1 Pa. Following in-detail theoretical analysis reported by Del Villar et al. [27], when *n* of the film increases, RI sensitivity of the LMR device follows. Since experimentally measured RI sensitivity increases with *p* in the discussed case (Figure 5C) one can expect, that *n* of ITO also increases with *p*. Thus, also following results shown in Figure 4B, when *p* increases the *N* drops. The *N* is a material property dependent on conditions of growth and the ITO film and its crystallinity. The crystallinity is strongly influenced by energy of impinging particles building the film [45, 46]. The energy of sputtered species is depleted in the plasma volume due to collisions with neutral gas atoms (Ar in our case). The energy depletion is proportional to the total number of gas atoms, and then can be considered as proportional to *p* [47]. The film prepared at higher *p* usually suffers from lower level of crystallinity, i.e., are typically amorphous [48]. Amorphous and polycrystalline structure of the film typically correspond to low mobility of charge carriers [49]. Hence, we can assume that rather amorphous ITO films prepared at higher *p* with lower *N* results in well-pronounced LMR and its high shift with applied *E* as shown in Figure 4B.

On top of optical properties, the *p* has also an impact on electrical properties of ITO. Tailoring of both electrical and the optical properties of the ITO film is important for two-domain simultaneous interrogation. Resistance (*R*) of the active part (25 mm) of the ITO-LMR has been measured directly on the optical fiber surface by an easy 2-pin method and the results for various *p* are shown in Figure 5D. It can be seen, that for *p* < 0.3 Pa the *R* does not exceed single kΩ, when for higher *p* (up to 1 Pa) it significantly increases. In general, *R* corresponds to *N* [21]. Furthermore, carrier mobility is typically higher in crystalline structures with larger grain domains [49] which preferential growth appears at lower *p*. It is documented in [29] that grain size and the level of crystallinity with preferential facet [*h*00] is strongly influenced by *p*. Vice versa for higher *p* smaller grains tend to growth, i.e., the deposited structure is expected to be polycrystalline or even amorphous, with reduced mobility of carriers that are scattered at boundaries of small grains. The results shown in Figure 5D fit well to the frame given above: the *R* increases significantly for *p* > 0.3 Pa, where lower level of crystallinity is expected. Thus, high *n* and high *R* of the ITO film correspond to low *N* that can be achieved for elevated *p*.

**Figure 6:**
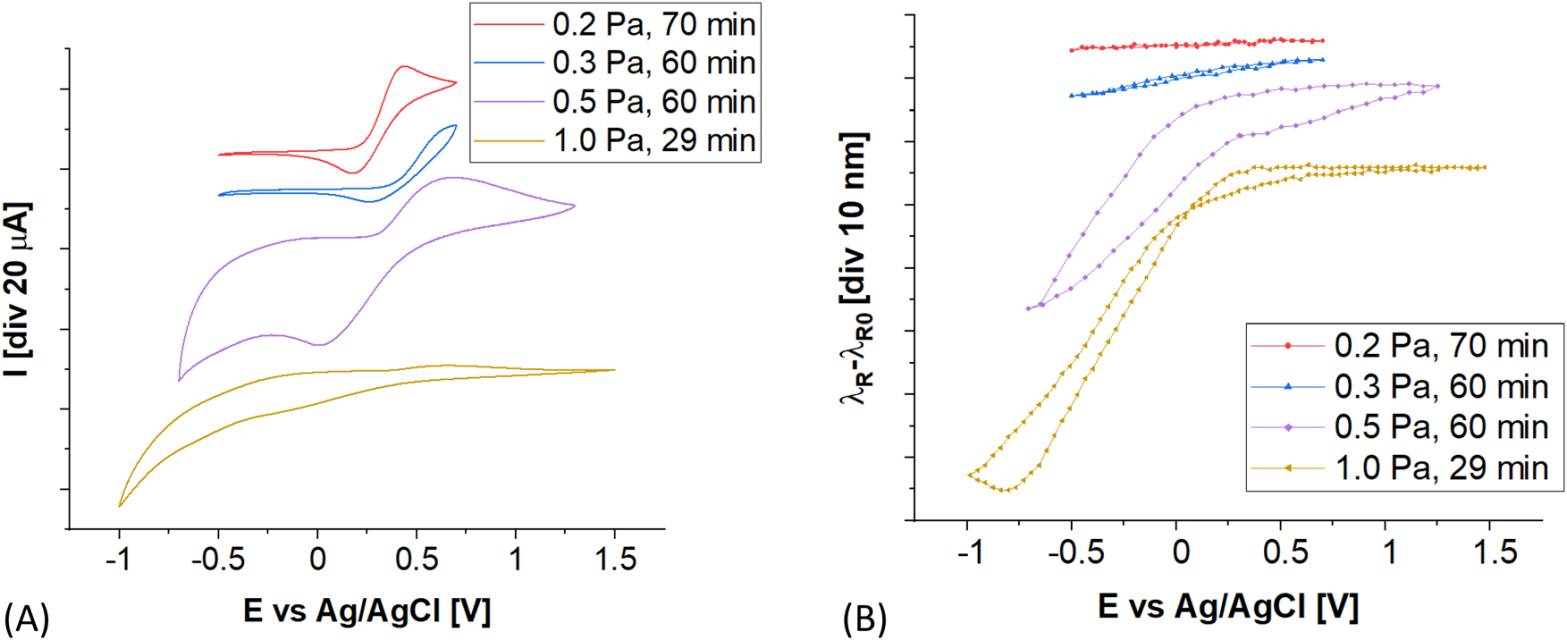
(A) EC and (B) optical responses received for ITO-LMR used as a working electrode, when various ITO deposition *p* are considered. The analysis was performed in 0.1 M PBS and 1mM 1,1′-ferrocenedimethanol as a redox probe. Scan rate was set to 20 mV/s.

The effect of electro-optical modulation of the ITO-LMR is discussed next. In Figure 6A plots obtained during CV scans are shown for ITO-LMR samples received at different *p*. The *E* was applied between the working (ITO film) and reference electrode, and an electrolyte containing redox probe was used. The presence of the probe has made current peaks observable for certain *E* values, that correspond to reduction and oxidation reactions. These peaks can be clearly identified for ITO deposited at low pressure, e.g., *p* = 0.2 Pa. These are the films with higher crystallinity [29], and subsequently higher *N*, and expected higher charge carrier mobility. It should be reminded here, that *N* (in particular *N*_acc_) may be further increased by the *E* applied to ITO (see Figure 2A). Due to enhanced *N*, the Fermi level of ITO rises and enables the exchange of the electrons with the electrolyte. In turn, when ITO films are prepared at higher pressures *p* > 0.2 Pa (Figure 6A), the redox current peaks tend to spread in the *E* domain and finally vanish, what can be clearly seen for *p* = 1 Pa. For the case of *p* > 0.3 Pa the scanning range of *E* has been increased, but even for these conditions the peaks can be hardly seen. It fits well with our previous statements that polycrystalline films with reduced mobility and *N* are preferentially deposited at higher *p*.

Optical responses of the ITO-LMR were recorded in parallel to CVs. Figure 6B shows relative shift of the *λ*_R_ with *E*. The *λ*_R_ was referred to initial measurements in the electrolyte when no *E* was applied (*λ*_R0_). For electrochemically-active samples, i.e., those with appearing redox current peaks deposited at *p* < 0.3 Pa, only very limited modulation effect is observed. The LMR modulation becomes effective for negative *E* and *p* > 0.3 Pa. In the investigated *p* range the modulation effectiveness reaches its maximum for *p* = 1 Pa, where d*λ*_R_/d*E* exceeds 40 nm/V. At these conditions the *N* seems to be the lowest in the investigated *p* range.

## 4 Conclusions

Former works reported that optical fiber structures coated with ITO can be successfully employed for sensing, including label-free biosensing, when LMR effect is obtained. Electrically conductive ITO can be also applied as a working electrode in electrochemical sensing systems. The presented research gives understanding of physical phenomena taking place in ITO thin film when it acts as a transducer for double-domain (optical and electrochemical) sensing concept. Both interrogation schemes work well separately, but their mutual combination for simultaneously active LMR and electrochemical sensing is challenging and requires precise tailoring of ITO film properties. We have clearly shown that properties expected for efficient optical and electrochemical interrogation are practically opposite. The properties can be tuned by means of input parameters during magnetron deposition. We found that charge carrier density corresponding to crystallinity of ITO is a critical parameter responsible for double-domain interrogation. Well-pronounced LMR peak with high *λ*_R_ shift occurs if the carrier density is low, but at these conditions when a negative potential is applied to ITO the charges effectively form the accumulation layer. The formation is macroscopically results in reduction of the ITO’s refractive index and allows for electro-optical modulation. On the other hand, effective electrochemical processes need ITO tailored toward high density of charge carriers. This is practically achieved in crystalline ITO films with larger grain domains where the carriers travel along the grains with reduced scattering at the grain boundaries. Finally, we can conclude that ITO gives rather narrow window of opportunities for simultaneous ITO-LMR and electrochemical interrogation, since introduced interrogating principles have competing conditions. Despite it, magnetron sputtering is a versatile deposition tool that enables precise ITO film tailoring to find (at least partially) the optimum conditions for simultaneous detection.

Results reported here may be treated as a starting point for work on other, alternative to ITO, semiconducting materials. These materials should uphold dual-domain applications serving as LMR supporting film and electrochemical electrode at the same time. However, it must be noted that as long as LMR can be obtained with a broad selection of thin film materials, very few materials may show suitable bandgap to offer electrochemical activity, as well as chemical and mechanical stability in the experimental conditions. Modulation frequency maximization following free charge carrier mobility or decreasing the film deposition costs motivate the exploration of other materials for dual-domain sensing. Properties of an alternative film may be tailored during the magnetron sputtering, as in case of ITO, by deposition pressure, but also by many other deposition parameters may be studied for this purpose.
